# Multidrug-resistant *Pseudomonas aeruginosa* in ICU patients and hospital surfaces: β-lactamase burden, biofilm formation and clonal spread

**DOI:** 10.1007/s10096-026-05457-w

**Published:** 2026-03-21

**Authors:** Marcos Eduardo Passos da Silva, Luccas Manoel de Melo Suica, Renata Santos Rodrigues, Márlon Grégori Flôres Custódio, Valcimar Batista Ferreira, Leilane da Silva Pontes, Ivson Cassiano de Oliveira Santos, Bruno Rocha Pribul, Núcia Cristiane da Silva Lima, Izabelly Vitória Gotara Ramos, Anjo Gabriel Carvalho, Mayra Gyovana Leite Belém, Rosimar Pires Esquerdo, Ana Paula D’Alincourt Carvalho Assef, Najla Benevides Matos

**Affiliations:** 1 Oswaldo Cruz Foundation – Rondônia, Porto Velho, Rondônia Brazil; 2 Experimental Biology Post-Graduate Program (PGBIOEXP), Federal University of Rondônia, Porto Velho, Rondônia Brazil; 3 Tropical Medicine Research Center (CEPEM), Rondônia Porto Velho, Brazil; 4 Hospital Infection Research Laboratory (LAPIH), Oswaldo Cruz Institute (IOC), Rio de Janeiro, Rio de Janeiro, Brazil; 5 Evandro Chagas National Institute of Infectious Diseases – INI/FIOCRUZ, Rio de Janeiro, Brazil

**Keywords:** *Pseudomonas aeruginosa*, Multidrug resistance, β-lactamases, Biofilm, Molecular Typing

## Abstract

**Introduction:**

*Pseudomonas aeruginosa* is one of the leading agents of Healthcare-Associated Infections (HAIs), especially in Intensive Care Units (ICUs), where its intrinsic resistance, biofilm-forming ability, and acquisition of β-lactamase genes contribute to therapeutic failure and increased morbidity. Understanding the resistance mechanisms and clonal dynamics of this pathogen in clinical and hospital environments is essential for effective infection control.

**Results:**

Overall, 30.5% of the isolates were classified as MDR, with higher non-susceptibility rates to imipenem (41.2%) and meropenem (35.6%). Moderate/strong biofilm formation was observed in 77.3% of the isolates. A high prevalence of β-lactamase genes was detected, particularly *blaCTX-M* (38.8%), *blaTEM* (29.1%), *blaGES* (13.9%), and *blaSHV* (4.1%), whereas carbapenemase genes were infrequent (*blaVIM*, *blaKPC*, *blaIMP*; 1.8%). The *blaCTX-M* gene was significantly associated with MDR profiles and resistance to cefepime and ceftazidime. PFGE identified 16 clonal groups among 28 isolates, indicating transmission among patients, hospital surfaces, and different hospitals. MLST revealed 16 sequence types, including four novel STs, with predominance of ST3079.

**Methods:**

A total of 216 *P. aeruginosa* isolates obtained from hospitalized patients and hospital surfaces in three public hospitals in Porto Velho, Western Amazon, Brazil, were analyzed. Antimicrobial susceptibility was determined according to CLSI guidelines. Biofilm formation was evaluated in 96-well microplates. β-lactamase and carbapenemase genes were detected by PCR. Genetic relatedness was investigated by PFGE and MLST in multidrug-resistant (MDR) or carbapenem-resistant isolates.

**Conclusion:**

*P. aeruginosa* from ICU patients and hospital surfaces showed high multidrug resistance, strong biofilm production, and widespread β-lactamase genes, particularly *blaCTX-M*, a key determinant of the MDR phenotype. Clonal dissemination across hospitals and sources highlights the need for strengthened infection control, continuous molecular surveillance, and antimicrobial stewardship.

**Supplementary Information:**

The online version contains supplementary material available at 10.1007/s10096-026-05457-w.

## Introduction

Healthcare-Associated Infections (HAIs) are among the major global public health challenges, as they are responsible for prolonging the length of hospital stay for admitted patients, thereby impacting morbidity and mortality rates [1, 2]. By definition, HAIs are infections acquired after patient admission to the hospital setting, with clinical manifestations occurring during or after the hospitalization period [3].

The First Global Report on Infection Prevention and Control released by the World Health Organization (WHO) highlighted that, among every 100 patients hospitalized with acute conditions, 7 in high-income countries and 15 in low-income countries will acquire some type of HAI, with an average of 1 in 10 progressing to death. Among the main microorganisms responsible for HAIs are bacteria, including *Pseudomonas aeruginosa* [4].

*P. aeruginosa* is a ubiquitous Gram-negative bacillus (GNB) with a high capacity for adaptation and can be found in a wide range of environments, including soil, water, plants, vegetables, animals, and hospital settings [5]. It is one of the leading pathogens responsible for hospital-acquired infections, capable of causing pneumonia, surgical site infections, urinary tract infections, and septicemia. It is estimated that this bacterium accounts for more than 7% of all HAIs and exhibits a colonization prevalence exceeding 50% among hospitalized patients [6, 7].

Another critical concern regarding *P. aeruginosa* is the increasing number of strains exhibiting antibiotic resistance, which has significantly impacted morbidity and mortality rates in intensive care units (ICUs) [7]. In 2024, the World Health Organization (WHO) updated the list of priority bacterial pathogens for public health to support research and the development of strategies to prevent the progression of antimicrobial resistance, classifying *P. aeruginosa* as a high-priority pathogen due to its resistance to carbapenems [8].

Carbapenems are the main antibiotics used to treat infections caused by Gram-negative bacilli (GNB) in intensive care units (ICUs), as they exhibit a broad spectrum of activity, high efficacy, and safety in administration. However, the increasing resistance to these agents has limited the therapeutic arsenal against infections caused by GNB, particularly *P. aeruginosa* [9, 10].

*P. aeruginosa* possesses multiple mechanisms of antibiotic resistance, which may be associated with membrane permeability, such as the production of efflux pumps and reduced porin expression, in addition to the production of enzymes that hydrolyze and inactivate antibiotics, including extended-spectrum β-lactamases (ESBLs) and carbapenemases [11, 12].

ESBLs and carbapenemases are enzymes encoded by genes that can be acquired via plasmid-mediated transfer, thereby increasing resistance potential and facilitating dissemination among bacteria. ESBLs are a predominant cause of β-lactam resistance, conferring the ability to hydrolyze penicillins, extended-spectrum cephalosporins, and aztreonam, with CTX-M, TEM, SHV, and GES being the main types reported in *P. aeruginosa* [13–15]. Regarding carbapenemases, these enzymes have a broad spectrum of activity, with hydrolytic action against carbapenems and most other β-lactams. Globally, *P. aeruginosa* exhibits a wide diversity of reported carbapenemases; in Latin America, KPC, IMP, VIM, SPM, and NDM are the most frequently observed [16–18].

Another factor associated with antimicrobial resistance in *P. aeruginosa* is biofilm formation. Biofilms are responsible for approximately 80% of chronic bacterial infections, contributing to prolonged hospital stays and adversely affecting morbidity and mortality rates [19, 20]. *P. aeruginosa* is a biofilm-forming organism, which enables resistance to adverse environmental conditions, enhances host colonization, and renders antimicrobial agents less effective against the pathogen [21, 22].

The implementation of molecular surveillance and typing of bacterial pathogens enables the characterization and monitoring of clinically relevant strains, particularly through the use of gold-standard techniques such as Pulsed-Field Gel Electrophoresis (PFGE) and Multilocus Sequence Typing (MLST). Both methods are essential for epidemiological surveillance and outbreak investigations, as they provide critical insights into bacterial population dynamics [23, 24].

In this context, the present study aimed to determine the antimicrobial susceptibility profile, identify genes associated with antimicrobial resistance and biofilm formation, and assess the clonal relationships of *Pseudomonas aeruginosa* isolates obtained from hospitalized patients and hospital surfaces in intensive care units (ICUs). The objective was to generate evidence-based data to support the adoption of new strategies for infection control and prevention of the dissemination of this pathogen, thereby reducing morbidity and mortality rates within ICUs.

## Materials and methods

### Sample collection and Processing

**Fig. 1:**
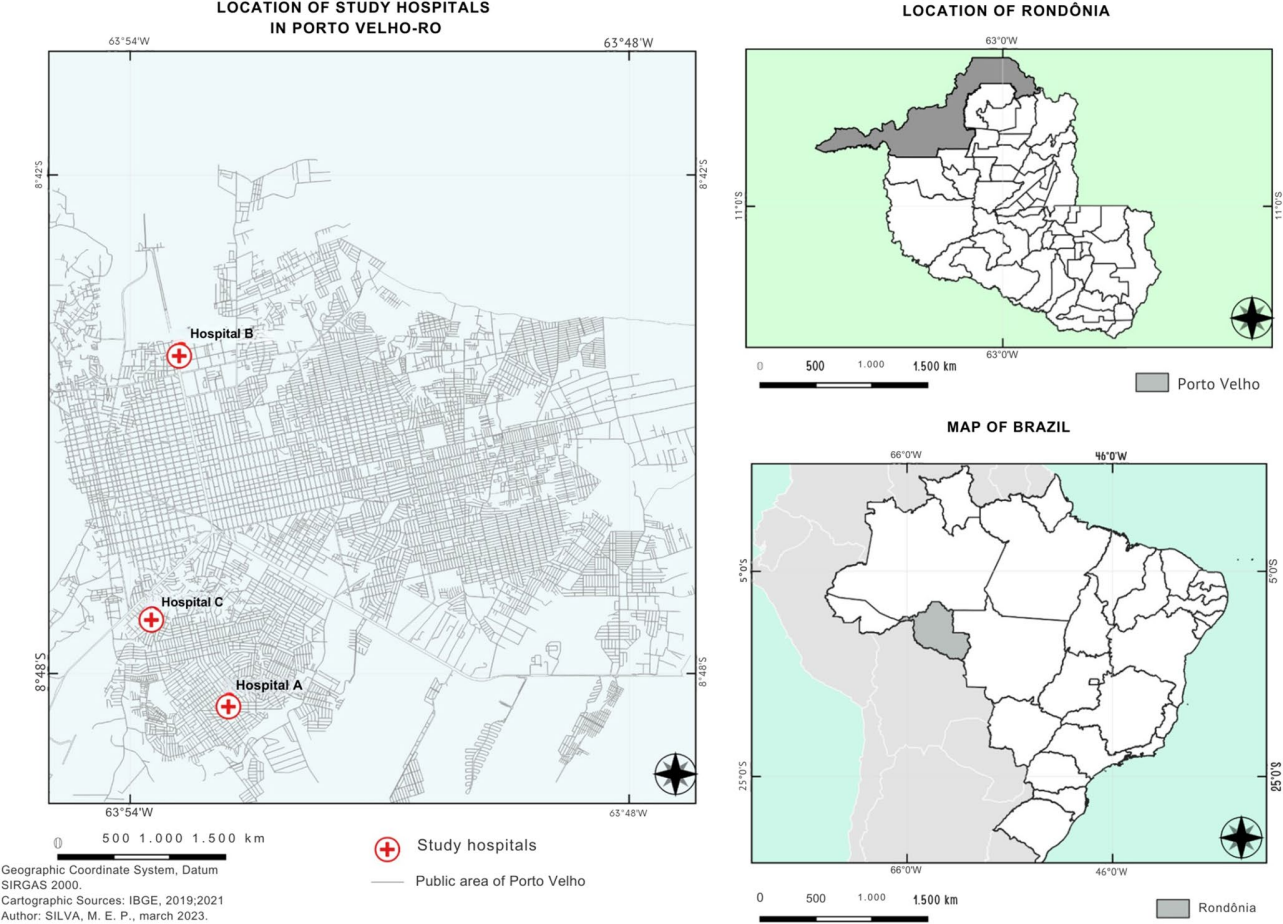
Geolocation of the study region and participating hospital. Source: the author

Samples were collected between December 2017 and February 2018, and between December 2018 and January 2019, from the adult ICU of three public hospitals (referred to in the study as Hospital A, Hospital B, and Hospital C) located in Porto Velho, Rondônia, Brazil (Fig. 1). Clinical samples (axilla, oral cavity, blood, tracheostomy, wound secretion, and urine) were obtained from hospitalized patients, as well as samples from hospital surfaces (air-conditioning systems, beds, sinks, faucets, wound secretions, and mechanical ventilation equipment).

After collection, the samples were transported to the Microbiology Laboratory of the Oswaldo Cruz Foundation in Rondônia (FIOCRUZ-RO) for processing, isolation, identification, and characterization of the microorganisms of interest.

This study was approved by the Research Ethics Committee of the Center for Research in Tropical Medicine, Porto Velho, Rondônia, Brazil (protocol no. 2.368.951, November 2017).

### Bacteriology and isolation

Urine and blood samples were directly inoculated onto selective and differential culture media. Samples collected using *swabs* were initially inoculated into Luria–Bertani (LB) broth (Kasvi^®^, Spain) and, after 24 h of incubation, were subcultured onto solid media. The culture media used for all samples were Blood Agar (BA; HiMedia^®^, India), MacConkey Agar (MC; FirstLab^®^, Brazil), CLED Agar (CL; Kasvi^®^, Spain), and Cetrimide Agar (CT; Biolog^®^). Plates were incubated in a bacteriological incubator at approximately 37 °C for 18–24 h.

Colonies suggestive of *Pseudomonas aeruginosa* were subjected to genomic DNA extraction using the phenol–chloroform method, followed by amplification of the 16 S rRNA gene by conventional PCR, as described by Arruda (2017) [25]. The amplified products were purified using the QIAquick Gel Extraction Kit (QIAGEN^®^, Germany), according to the manufacturer’s instructions. Purified DNA was quantified using a NanoDrop 1000^®^ spectrophotometer (Thermo Scientific^®^, USA) and subsequently sequenced using the Sanger method. Sequence analysis and consensus sequence generation were performed using BioEdit Sequence Alignment Editor (version 7.0), and species identification and confirmation were conducted using the Basic Local Alignment Search Tool (BLAST) database.

### Antimicrobial susceptibility testing (AST)

AST was performed using the Kirby–Bauer disk diffusion method on Mueller–Hinton agar (MH; Kasvi^®^, Italy), in accordance with the 2022 recommendations of the Clinical and Laboratory Standards Institute (CLSI). The following antimicrobial agents were tested: piperacillin–tazobactam (PPT, 100/10 µg), ceftazidime–avibactam (CZA, 30/20 µg), ceftolozane–tazobactam (C/T, 30/10 µg), ceftazidime (CAZ, 30 µg), cefepime (CPM, 30 µg), aztreonam (ATM, 30 µg), imipenem (IPM, 10 µg), meropenem (MER, 10 µg), gentamicin (GEN, 10 µg), tobramycin (TOB, 10 µg), amikacin (AMI, 30 µg), ciprofloxacin (CIP, 5 µg), and levofloxacin (LVX, 5 µg).

For susceptibility testing to polymyxin B, the broth microdilution method was employed to determine the minimum inhibitory concentration (MIC) using the POLICIMBAC kit (Probac^®^, Brazil). *Pseudomonas aeruginosa* ATCC^®^ 27,853 was used as the quality control strain.

The criteria for classifying the isolates as multidrug-resistant (MDR) in relation to the tested antimicrobial agents followed the definition proposed by Magiorakos et al. (2012) [26].

### Biofilm formation assay

The ability of the isolates to form biofilms was evaluated in triplicate, following the methodology described by Alvim (2019) [27], with minor modifications, using 96-well polystyrene microplates. *P. aeruginosa* isolates were cultured in Luria–Bertani (LB) broth (Kasvi^®^, Spain) under agitation at 120 rpm in an orbital shaker at approximately 37 °C for 18–24 h. After incubation, the cultures were diluted 1:20 in sterile LB, and 200 µL of each suspension was dispensed in triplicate into the wells of polystyrene microplates (Costar^®^, USA), followed by incubation at approximately 37 °C for 24 h. The plates were then washed twice with 200 µL of distilled water to remove the LB medium and non-adherent cells.

Adherent bacterial cells were stained with 100 µL of 0.1% (w/v) crystal violet solution (Laborclin^®^, Brazil) for 5 min. Excess stain was removed by rinsing with distilled water, and the bound dye was solubilized with 95% ethanol (Neon^®^, Brazil). Biofilm biomass was quantified by measuring absorbance at 570 nm (optical density [OD]) using a spectrophotometer (BioTek Epoch^®^, USA). *Escherichia coli* ATCC^®^ 25922 was used as the negative control, whereas *P. aeruginosa* ATCC^®^ 27853 served as the positive control.

Results were analyzed based on the mean OD values of the triplicates for each isolate. The cutoff value was defined as the mean OD of the negative control plus three standard deviations. Isolates with OD values equal to or below this cutoff were classified as weak/non-biofilm producers, while those with OD values above the cutoff were categorized as moderate/strong biofilm producers.

### Detection of resistance genes by polymerase chain reaction (PCR)

Conventional PCR was employed for the identification of antimicrobial resistance genes. PCR reaction kits from Sinapse Inc^®^ and Ludwig Biotec^®^ were used, and amplification cycles were carried out in a Veriti™ 96-Well Thermal Cycler (Applied Biosystems^®^). The primers used in this study are listed in Table 1. Reference strains and the amplification conditions for each assay are described in Supplementary Materials 1 and 2, respectively.

**Table 1:** Primers used for the detection of resistance genes in *P. aeruginosa*

Genes	Primers (5’ →3’)	Size (bp)	Reference
*blaCTX-M*	CTX-M **F** - TTTGCGATGTGCAGTACCAGTAA CTM-M **R** - CGATATCGTTGGTGGCCATA	544	[28]
*blaSHV*	SHV **F** – ATGCGTTATATTCGCCTGTG SHV **R** - TGCTTTGTTATTCGGGCCAA	747	[29]
*blaTEM*	TEM **F** – TCGCCGCATACACTATTCTCAGAATGA TEM **R** - ACGCTCACCGGCTCCAGATTTAT	593	
*blaGES*	GES **F** – CTATTACTGGCAGGGATCG GES **R** - CCTCTCAATGGTGTGGGT	594	[30]
*blaKPC*	KPC **F** - TCGCTAAACTCGAACAGG KPC **R** – TTACTGCCCGTTGACGCCCAATCC	785	
*blaNDM*	NDM **F** - TTGGCCTTGCTGTCCTTG NDM **R** - ACACCAGTGACAATATCACCG	82	
*blaVIM*	VIM **F** – GTTTGGTCGCATATCGCAAC VIM **R** - AATGCGCAGCACCAGGATAG	382	
*blaOXA-48*	OXA-48 **F** – TGTTTTTGGTGGCATCGAT OXA-48 **R** - GTAAARATGCTTGGTTCGC	177	
*blaIMP*	IMP-1 **F** – CTACCGCAGCAGAGTCTTTG IMP-1 **R** - AACCAGTTTTGCCTTACCAT	587	[31]
*blaSPM*	SPM **F** – CCTACAATCTAACGGCGACC SPM **R** - TCGCCGTGTCCAGGTATAAC	650	[32]
mcr-1	CLR-5 **F** – CGGTCAGTCCGTTTGTTC CLR-5 **R** - CTTGGTCGGTCTGTA	308	[33]

bp = base pairs

### Pulsed-field gel electrophoresis (PFGE)

PFGE was performed exclusively on isolates classified as multidrug-resistant (MDR), extensively drug-resistant (XDR), or pandrug-resistant (PDR), as well as on isolates exhibiting resistance to carbapenems. The experiments were conducted at the Laboratory for Research in Hospital Infection (LAPIH), Oswaldo Cruz Institute, Rio de Janeiro, Brazil.

The isolates were reactivated on nutrient agar at 37 °C for 18–24 h. Pure colonies were suspended in BSC solution (0.5 M EDTA, 1 M Tris, pH 8) to a turbidity of approximately 3 on the McFarland scale and vortexed. The suspension was then mixed with proteinase K (50 mg/mL) and 1% agarose containing 20% SDS, and dispensed into molds to obtain agarose plugs. The plugs were subjected to cell lysis in Tris–EDTA–sarcosyl solution containing proteinase K at 46 °C for 2 h, followed by sequential washing with ultrapure water and TE buffer, and subsequently stored under refrigeration.

For the pre-restriction step, one-third of each agarose plug was incubated with enzyme buffer at 4 °C for 30 min. During the restriction phase, the plugs were digested with the restriction enzyme BcuI (20 U/µL) at 37 °C for 3 h. After washing with 0.4× TBE buffer, the plugs were subjected to pulsed-field gel electrophoresis in a 1.1% agarose gel (CHEF-DRIII system, Bio-Rad), using a ramped pulse time of 5–35 s for 17 h at 6 V/cm, with a 120° angle at 14 °C. Pulse Marker (Sigma^®^) was used as the molecular weight marker.

The gels were stained with GelRed™ and visualized under UV light, with photographic documentation. DNA profile analysis was performed using BioNumerics software version 6.6 (Applied Maths), applying the Dice similarity coefficient with optimization and tolerance settings of 1.5%. Isolates exhibiting a similarity coefficient ≥ 85% were considered to belong to the same clonal group, according to the criteria proposed by Tenover et al. (1995): indistinguishable patterns (100% similarity), closely related (2–3 band differences), and possibly related (4–6 band differences).

### Multilocus sequence typing (MLST)

MLST was performed only on isolates classified as multidrug-resistant (MDR), following the same criteria applied for PFGE. PCR amplification of the seven housekeeping genes was carried out according to Curran (2004) [34], using standardized conditions: an initial denaturation at 94 °C for 3 min; 35 cycles at 96 °C for 1 min, 55 °C for 1 min, and 72 °C for 1 min; and a final extension at 72 °C for 7 min. PCR products were purified using the ExoSAP-IT™ kit (Thermo Fisher Scientific^®^) and sequenced by the Sanger method at Fiocruz-Bahia.

Sequence assembly and analysis were performed using the TRACY software, employing the Pearl application to correct inconsistencies and generate consensus contigs. Alleles and sequence types (STs) were assigned using the *Pseudomonas aeruginosa* PubMLST database (pubmlst.org). Phylogenetic relationships among STs were evaluated using the eBURST software, with isolates differing by only one allele considered to belong to the same clonal complex.

### Statistical analyses

The results were tabulated using Microsoft Excel spreadsheets, and statistical analyses were performed using GraphPad Prism version 5.0 and the R software environment. Nonparametric tests, including Fisher’s exact test and odds ratio analysis, were applied. Statistical significance was defined as a p value < 0.05.

## Results

Part of the isolates obtained from the oral cavity of patients had been previously published by this research group in Silva (2025); in the present study, these isolates were included and subjected to additional analyses.

A total of 216 *Pseudomonas aeruginosa* isolates were obtained. Of these, 50.9% (110/216) originated from Hospital A, 34.7% (75/216) from Hospital C, and 14.3% (31/216) from Hospital B. Regarding the source, 75% (162/216) were isolated from patients and 25% (54/216) from hospital surfaces. Among patient-derived isolates, the main sampling sites were the oral cavity (48.1% [78/162]), tracheostomy (22.9% [37/162]), and axilla (14.8% [24/162]). Among hospital surfaces, isolates were most frequently recovered from sinks (40.7% [22/54]), faucets (35.2% [19/54]), and beds (20.4% [11/54]) (Table 2).

**Table 2:** Distribution of *Pseudomonas aeruginosa* according to hospitals and sampling sites in hospitalized patients and hospital surfaces

Samples (*n* = 216)	Hospitals					
	Hospital A		Hospital B		Hospital C	
	n	%	n	%	n	%
**Origin**						
Patients (*n* = 162; 75%)	88	54.3	19	11.7	55	34
Hospital surfaces (*n* = 54; 25%)	22	40.8	12	22.2	20	37
**Collection Site**						
**Patients** (*n* = 162)						
Axilla (14.8%)	5	20.8	7	29.2	12	50
Oral cavity (48.1%)	49	62.8	12	15.4	17	21.8
Blood (3.7%)	2	33.3	0	0	4	66.7
Wound secretion (3.7%)	0	0	0	0	6	100
Tracheostomy (22.9%)	21	56.7	0	0	16	43.3
Urine (6.8%)	11	100	0	0	0	0
**Hospital surfaces** (*n* = 54)						
Bed (20.4%)	2	18.3	2	18.3	7	63.4
Sink (40.7%)	7	31.8	5	22.7	10	45.5
Faucet (35.2%)	12	63.1	5	26.3	2	10.6
Floor (3.7%)	1	50	0	0	1	50

**Fig. 2:**
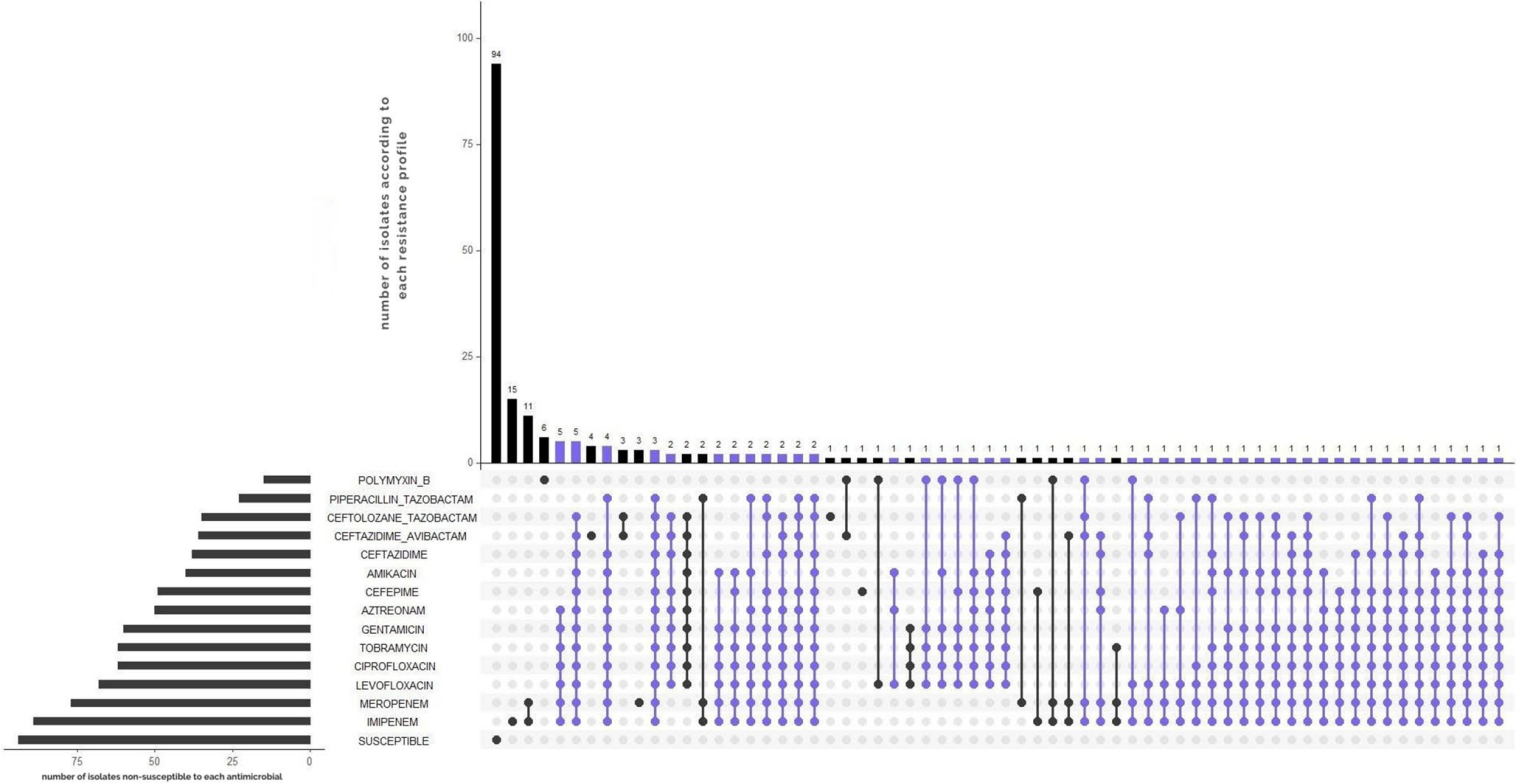
Antimicrobial susceptibility profile, non-susceptibility patterns, and multidrug resistance of *Pseudomonas aeruginosa* isolates. Legend: The horizontal bars on the left represent the number of isolates that were non-susceptible to each antimicrobial. Antibiotic resistance patterns are represented by the connected dots. Purple dots indicate MDR patterns, while black dots indicate non-MDR patterns. The vertical bars represent the number of isolates each non-susceptibility pattern.

The highest susceptibility rates were observed for polymyxin B (93% [201/216]), piperacillin–tazobactam (89.3% [193/216]), ceftolozane–tazobactam (83.7% [181/216]), and ceftazidime–avibactam (82.8% [179/216]). The highest rates of non-susceptibility were observed for imipenem (41.2% [89/216]), meropenem (35.6% [77/216]), levofloxacin (31.4% [68/216]), and ciprofloxacin (28.7% [62/216]). Resistance to polymyxin B was detected in 6.9% (15/216) of the isolates. Overall, 30.5% (66/216) of the isolates were non-susceptible to at least one antibiotic from three different classes and were therefore classified as multidrug-resistant (MDR) (Fig. 2).

**Fig. 3:**
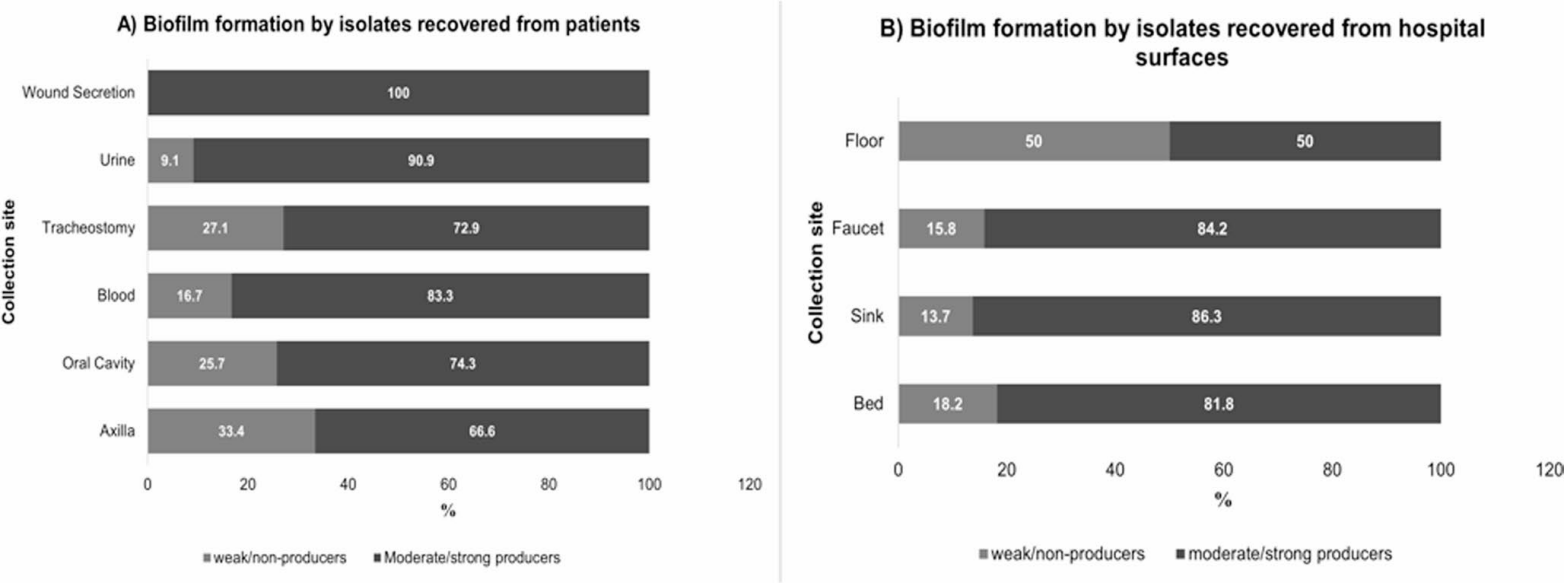
Biofilm-forming capacity of *Pseudomonas aeruginosa* isolates

Regarding biofilm formation, 77.3% (167/216) of the isolates were classified as moderate or strong biofilm producers. Among clinical isolates, 75.3% (122/162) exhibited this profile, with the highest frequencies observed in wound secretions (100% [6/6]), urine (90.9% [10/11]), and blood (83.3% [5/6]) (Fig. 3a). Among isolates recovered from hospital surfaces, 83.3% (45/54) were moderate or strong biofilm producers, particularly those obtained from sinks (86.3% [19/22]) and faucets (84.2% [16/19]) (Fig. 3b). An association analysis between biofilm formation and multidrug resistance among the isolates revealed a significant relationship (*p* < 0.0004; OR = 0.2027), with moderate/strong biofilm-producing isolates exhibiting a higher frequency of MDR.

The resistance genes detected and their frequencies are described in Table 3. The most prevalent gene was *blaCTX-M* (38.8% [84/216]), followed by *blaTEM* (29.1% [63/216]) and *blaGES* (13.9% [30/216]). The *blaTEM* gene was detected exclusively in clinical isolates. Carbapenemase genes were rarely identified: *blaVIM* (0.9% [2/216]), *blaIMP* (0.4% [1/216]), and *blaKPC* (0.4% [1/216]), all detected in patient-derived isolates. No isolate tested positive for *blaNDM*, *blaOXA-48*, or mcr-1.

**Table 3:** Distribution of detected genes in *Pseudomonas aeruginosa* according to sampling sites and collection locations

Samples (*n* = 216)	Genes													
	bla_CTX−M_		bla_TEM_		bla_GES_		bla_SHV_		bla_VIM_		bla_KPC_		bla_IMP_	
*n*	%	*n*	%	*n*	%	*n*	%	*n*	%	*n*	%	*n*	%	
**Origin**														
Patients (*n* = 162)	82	50.6	63	38.8	28	17.2	8	4.9	2	1.2	1	0.6	1	0.6
Hospital surfaces (*n* = 54)	2	3.7	0	0	2	3.7	1	1.8	0	0	0	0	0	0
**Collection site**														
**Patients** (*n* = 162)														
Axilla (*n* = 24)	7	29.1	5	20.8	0	0	5	20.8	0	0	0	0	1	4.2
Oral cavity (*n* = 78)	45	57.6	39	50	15	19.2	0	0	1	1.2	1	1.2	0	0
Blood (*n* = 6)	2	33.3	0	0	0	0	0	0	0	0	0	0	0	0
Wound secretion (*n* = 6)	2	33.3	2	33.3	0	0	0	0	0	0	0	0	0	0
Tracheostomy (*n* = 37)	17	45.9	12	32.4	6	16.2	3	8.1	0	0	0	0	0	0
Urine (*n* = 11)	9	81.8	5	45.4	7	63.3	0	0	1	9.0	0	0	0	0
**Hospital surfaces** (*n* = 54)														
Bed (*n* = 11)	2	18.1	0	0	0	0	0	0	0	0	0	0	0	0
Sink (*n* = 22)	0	0	0	0	2	9.0	1	4.5	0	0	0	0	0	0
Faucet (*n* = 19)	0	0	0	0	0	0	0	0	0	0	0	0	0	0
Floor (*n* = 2)	0	0	0	0	0	0	0	0	0	0	0	0	0	0

Isolates from the oral cavity accounted for the highest frequency of detected genes (101 genes), predominantly *blaCTX-M* (44.5% [45/101]) and *blaTEM* (38.6% [39/101]). Among hospital surface isolates, only five genes were identified, mainly *blaCTX-M* and *blaGES*, primarily in isolates recovered from sinks and beds.

**Fig. 4:**
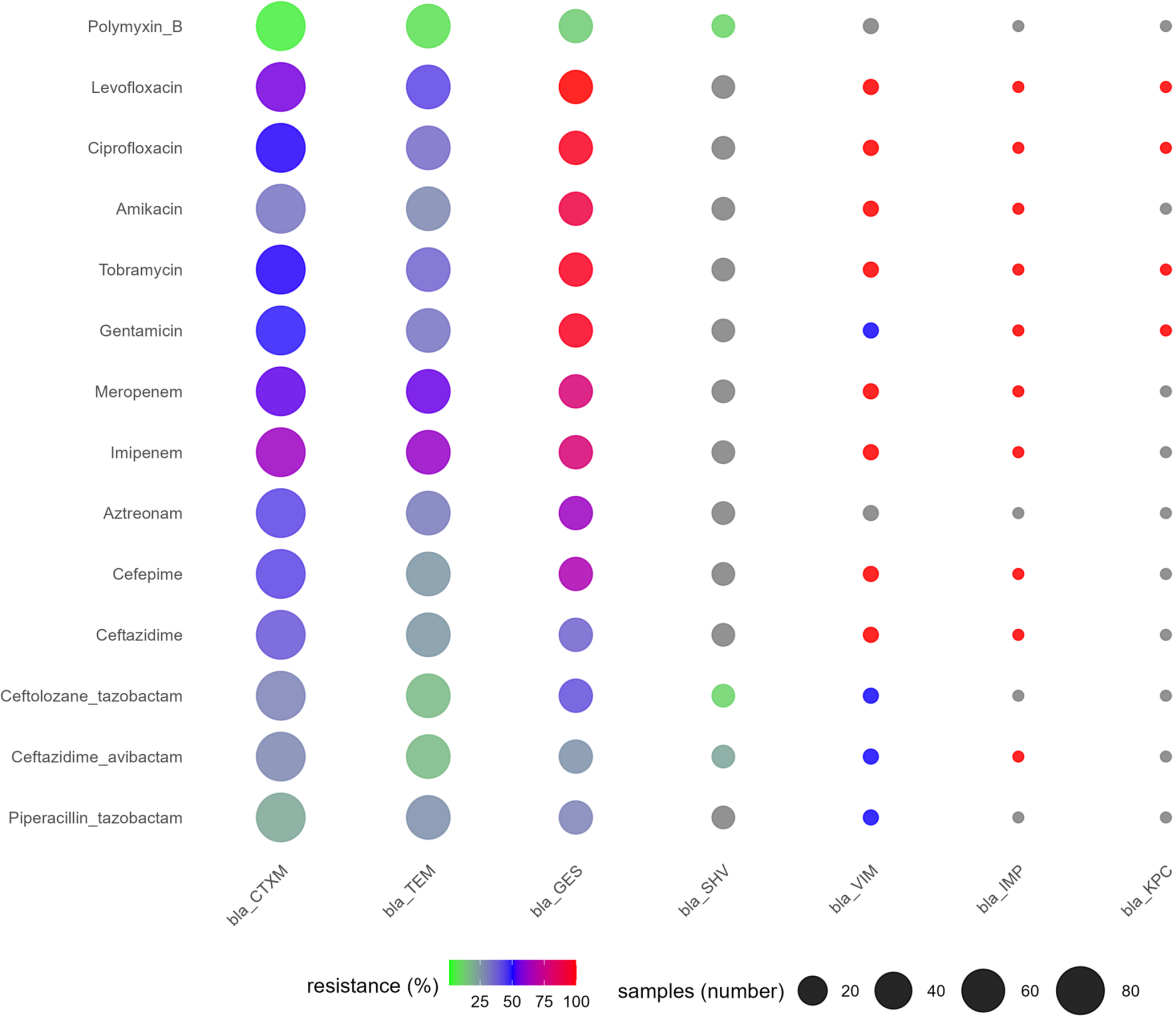
Non-susceptibility profile of isolates positive for the detected resistance genes

**Fig. 5:**
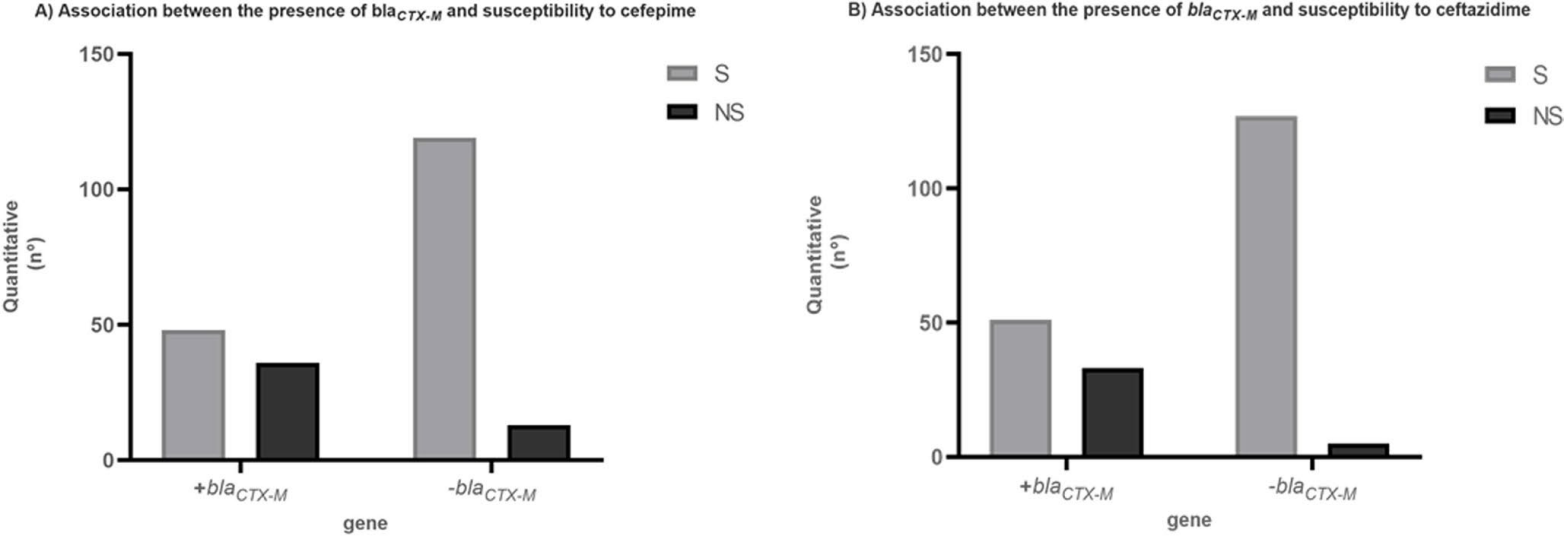
Association of *Pseudomonas aeruginosa* isolates carrying the *blaCTX-M* gene with resistance to cephalosporin antibiotics. S: Susceptible. NS: Non-susceptible. Statistical significance was considered at *p* < 0.05

**Fig. 6:**
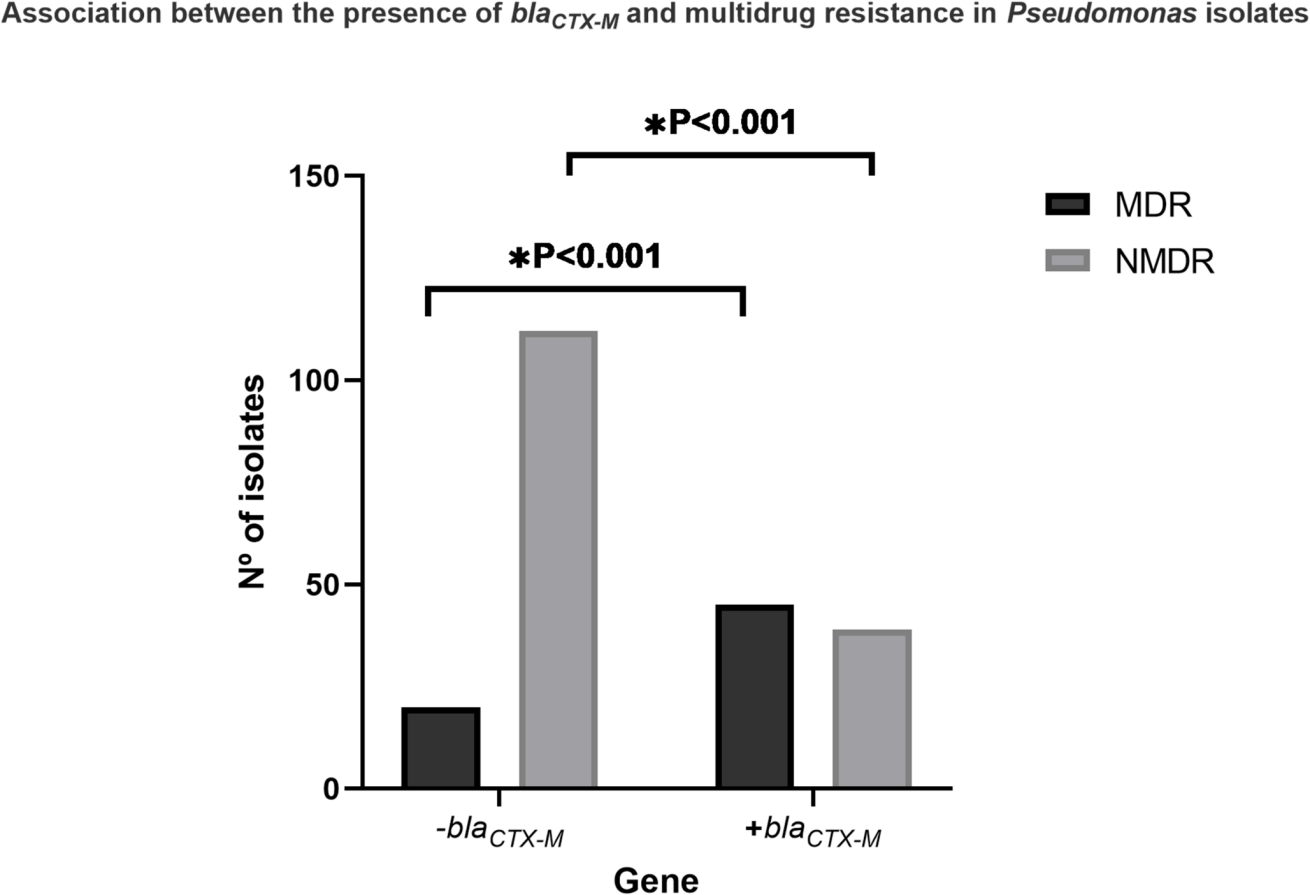
Association of *Pseudomonas aeruginosa* isolates carrying the *blaCTX-M* gene with the multidrug resistance profile. MDR: Multidrug-resistant isolates. NDMR: Non–multidrug-resistant isolates. Statistical significance was defined at *p* < 0.05

Isolates positive for *blaCTX-M* exhibited high rates of non-susceptibility to carbapenems (imipenem: 60.7% [51/84]; meropenem: 54.7% [46/84]) and to cephalosporins (cefepime: 42.8% [36/84]; ceftazidime: 39.2% [33/84]) (Fig. 4). A statistically significant association was observed between the presence of *blaCTX-M* and resistance to cefepime (*p* < 0.0001; OR = 0.1457), ceftazidime (*p* < 0.0001; OR = 0.0608), and classification as MDR (*p* < 0.001; OR = 0.1548) (Figs. 5 and 6).

**Fig. 7:**
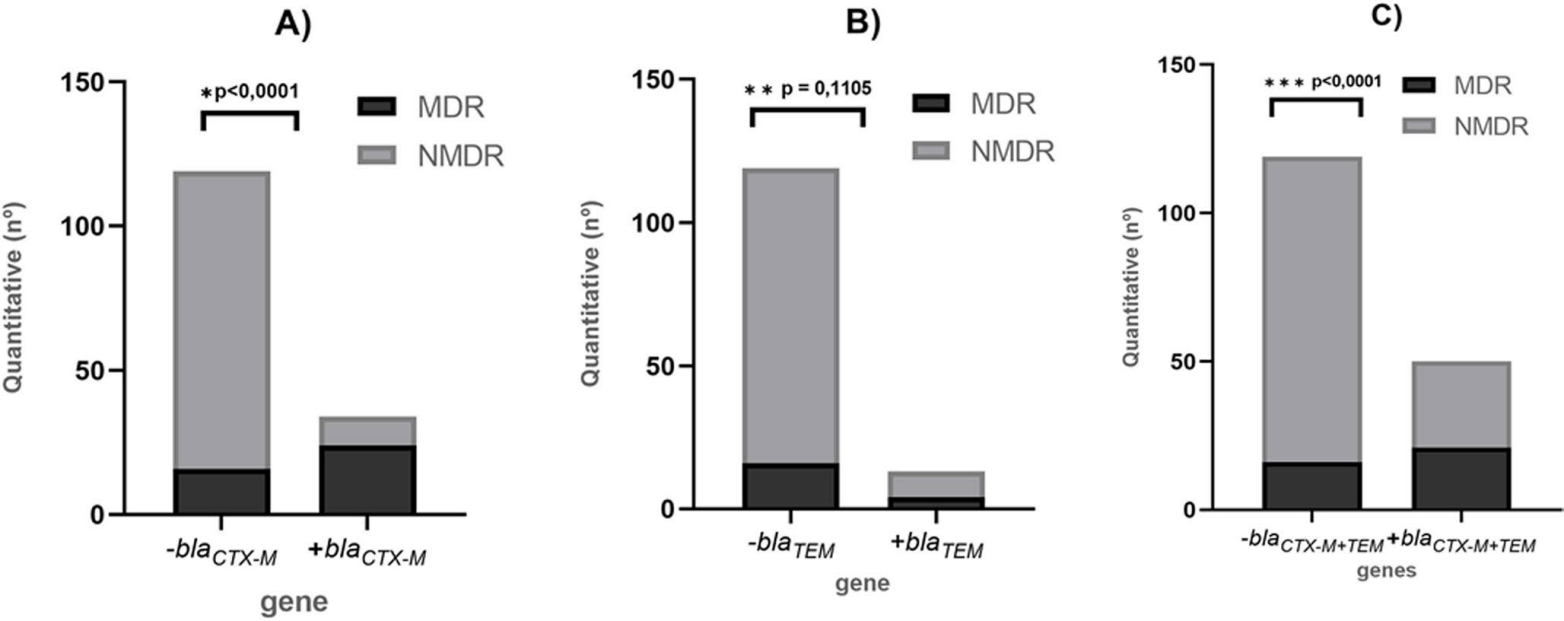
This data is mandatory. please provide

The *blaTEM* gene was the second most frequently detected, with higher rates of non-susceptibility to carbapenems (imipenem: 61.9% [39/63]; meropenem: 55.5% [35/63]), but with a lower impact on cephalosporins (25.3% [16/63]) (Fig. 4). Co-production of *blaCTX-M*/*blaTEM* was observed in 79.3% (50/63) of *blaTEM*-positive isolates, of which 42% (21/50) were classified as MDR. Isolates harboring *blaCTX-M* alone showed a significant association with MDR (*p* < 0.0001), whereas those harboring *blaTEM* alone did not show a significant association. Co-production of both genes remained associated with MDR, suggesting a greater influence of *blaCTX-M* on this phenotype (Fig. 7). The *blaGES* gene was detected in 13.9% (30/216) of the isolates, with 80% (24/30) exhibiting non-susceptibility to carbapenems and 93.3% (28/30) classified as MDR. Co-production with *blaCTX-M* and *blaTEM* was frequent. The *blaSHV* gene was identified in only nine isolates, which exhibited a broadly susceptible antimicrobial profile (Fig. 4).

**Fig. 8:**
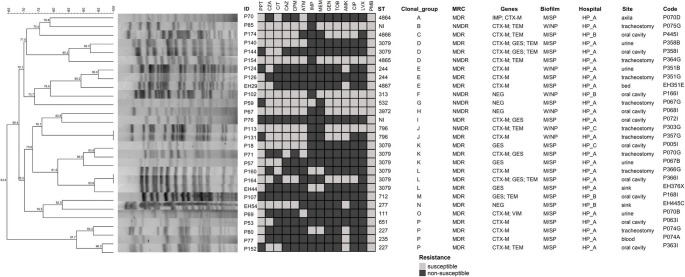
Dendrogram, antimicrobial susceptibility profile, resistance genes, and biofilm production of *Pseudomonas aerugino*sa isolates from hospitalized patients and hospital surfaces. **ID**: sample identification. **Code**: Patients (P) and hospital surfaces (EH). **ST**: Sequence Type; **IMP**: Imipenem; **MER**: Meropenem; **LVX**: Levofloxacin; **TOB**: Tobramycin; **CIP**: Ciprofloxacin; **GEN**: Gentamicin; **CAZ**: Ceftazidime; **CZA**: Ceftazidime–Avibactam; **CPM**: Cefepime; **AMK**: Amikacin; **C/T**: Ceftolozane–Tazobactam; **ATM**: Aztreonam; **PPT**: Piperacillin–Tazobactam. **MRC**: Multidrug resistance classification; **MDR**: Multidrug-resistant; **NDMR**: Non–multidrug-resistant. Genes: Resistance genes. **BP**: Biofilm production; **NI**: Not tested; **W/NP**: Weak/non-producer of biofilm; **M/SP**: Moderate/strong biofilm producer

For clonal analysis, 28 MDR or carbapenem-resistant isolates were selected. PFGE analysis grouped the isolates into 16 clonal groups (A–P), considering a similarity threshold of ≥ 85% (Fig. 8). Clonal group P was the most frequent (*n* = 4), including isolates from the same patient recovered from different anatomical sites, indicating endogenous dissemination. Other clonal groups also demonstrated dissemination across different sites, hospital surfaces, and distinct hospitals.

**Fig. 9:**
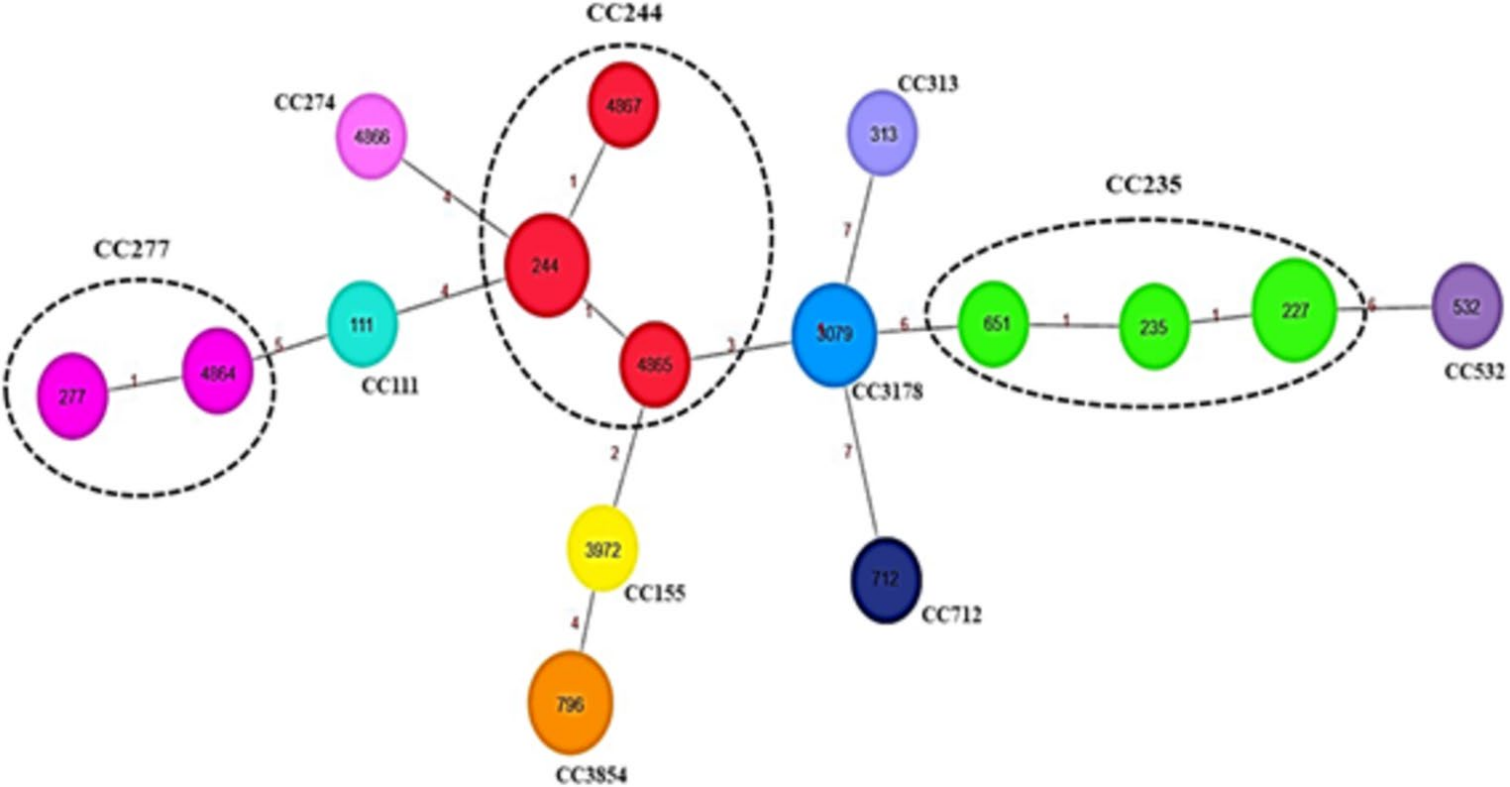
Distribution of clonal complexes of the sequence types (STs) of *Pseudomonas aeruginosa* isolates. Figure generated using the online PHYLOViZ software showing a minimum spanning tree (MST) representing the ancestral relationships among the 16 STs identified in the study. Each colored circle represents a sequence type (ST). The numbers on the lines connecting the circles indicate the number of allelic differences between STs. Dashed circles group STs belonging to the same clonal complex

MLST analysis identified 16 sequence types (STs) and 11 clonal complexes (CCs)[supplementary material 3]. The most prevalent ST was ST3079 (*n* = 8), followed by ST227, ST244, and ST796 (*n* = 2 each). Four novel STs were described (ST4864–ST4867). The most frequent CCs were CC3178, CC235, and CC244 (Fig. 9).

Integrated analysis of PFGE, MLST, antimicrobial susceptibility profiles, resistance genes, and biofilm formation demonstrated the circulation of MDR, biofilm-producing, ESBL-harboring clones among patients and hospital surfaces, including inter-hospital dissemination.

## Discussion

*Pseudomonas aeruginosa* is a pathogen of high relevance in nosocomial infections, being associated with bacteremia, surgical site infections, and pneumonia, particularly in immunocompromised individuals. Its ability to persist in hospital environments contributes to intrinsic resistance to antibiotics [35, 36].

The literature indicates that imbalances in the oral microbiota resulting from systemic diseases, immunosuppression, or medication use favor colonization by opportunistic pathogens such as *P. aeruginosa* [37, 38]. High rates of oral colonization have also been documented in patients after cardiac surgery [39], as well as in institutionalized older adults and mechanically ventilated patients, in whom the bacterium frequently colonizes dental plaque and endotracheal tubes, contributing to aspiration pneumonia [40, 41].

Tracheostomy was the second most frequent sampling site (22.9%), a proportion lower than that reported in other national and international studies [42, 43]. These findings reinforce the need for intensive oral hygiene care in intensive care units, a measure that has already been associated with reductions in ventilator-associated pneumonia and mortality [44, 45].

*P. aeruginosa* was also isolated from hospital surfaces, such as sinks, faucets, and beds, which are recognized as reservoirs of resistant microorganisms [46, 47]. Previous studies have demonstrated persistent environmental colonization and its association with dissemination to patients and healthcare professionals [48–50]. These findings reinforce the importance of effective disinfection practices and hand hygiene [51].

The antimicrobial resistance observed follows global trends. Data from the SENTRY program indicate higher rates of multidrug resistance in Latin America (41.1%) and an increasing trend toward carbapenem resistance [52]. Regional and temporal variations are widely reported [53–56], reinforcing the influence of indiscriminate antibiotic use, which was further intensified during the COVID-19 pandemic [57].

Despite carbapenem resistance, high susceptibility to β-lactam/β-lactamase inhibitor combinations was observed, particularly to ceftolozane–tazobactam and ceftazidime–avibactam, in agreement with other studies [58, 59]. The resistance rates identified were consistent with those reported in different regions [60–62]; however, in the present study, no association was observed between *blaGES* and resistance to these agents.

Biofilm formation was high (77.3%), an expected finding for *P. aeruginosa*, whose ability to form biofilms is a critical virulence factor and facilitates horizontal transfer of resistance genes [63–65]. International studies report biofilm formation rates ranging from 40% to 95%, further supporting the association between biofilm formation and carbapenem resistance [66–68]. In ICU settings, biofilm formation enhances persistence on medical devices and moist surfaces, contributing to recurrent colonization and increased tolerance to disinfectants and antimicrobial therapy [69, 70]. The observed association between moderate/strong biofilm formation and multidrug resistance is consistent with previous evidence indicating that biofilm growth enhances antimicrobial tolerance and promotes persistence in hospital environments. Furthermore, the association between combinations of resistance genes and increased biofilm production suggests that high-risk clones may simultaneously benefit from resistance and virulence traits, thereby contributing to their persistence and dissemination [71, 72].

ESBL-type β-lactamases predominated, particularly *blaCTX-M*, in agreement with international findings [73]. Co-production of *blaCTX-M* and *blaTEM* was frequent, a condition that, according to Idrees et al. (2023) and Tufa (2022), broadens the resistance spectrum [74, 75]. The elevated frequency of *blaCTX-M* observed may reflect the strong selective pressure exerted by extensive β-lactam use in hospital settings, which favors the expansion and persistence of ESBL-producing organisms. Genes of the CTX-M family are commonly associated with mobile genetic elements such as plasmids, integrons, and insertion sequences, facilitating horizontal transfer and rapid dissemination under antimicrobial pressure [76, 77]. In Brazil, CTX-M enzymes widely disseminated in clinical settings, highlighting their adaptive success and epidemiological relevance. Additionally, environmental reservoirs, including hospital effluents and aquatic ecosystems, may contribute to the maintenance and circulation of *blaCTX-M* in the Amazon region, reinforcing the importance of integrated surveillance strategies to limit its spread [78, 79].

The low detection of carbapenemases is consistente with reports indicating limited endemicity of certain carbapenemases genes in Brazil [80, 81]. Therefore, carbapenem resistance may be associated with secondary mechanisms, such as AmpC hyperproduction, porin loss, and efflux pump overexpression [82]. In *Pseudomonas aeruginosa*, reduced expression or mutational inactivation of the outer membrane porin OprD represents one of the most common mechanisms associated with imipenem resistance, limiting antibiotic uptake into the bacterial cell [83]. In parallel, overexpression of multidrug efflux systems, such as the MexAB-OprM pump complex, contributes to decreased susceptibility to multiple antimicrobial agents and plays a central role in multidrug resistance [84, 85]. Hyperproduction or structural mutations of the chromosomal AmpC β-lactamase further increase resistance by enhancing hydrolytic activity against β-lactams, including carbapenems and modern β-lactam/β-lactamase inhibitor combinations [21, 86]. Importantly, these mechanisms often coexist within the same strain, producing additive or synergistic effects that result in high-level resistance even in the absence of acquired carbapenemase genes [87]. Similar profiles have been described in non-carbapenemase-producing isolates, in which OprD loss, efflux pump overexpression, and AmpC upregulation act in concert to promote carbapenem resistance [88]. These adaptive strategies reflect the remarkable genomic plasticity of *P. aeruginosa* and may be favored under prolonged antimicrobial pressure in intensive care environments.

Clonal analysis (PFGE and MLST) revealed both intra- and inter-hospital dissemination. Clonal groups included isolates recovered from different anatomical sites of the same patient, indicating cross-colonization between anatomical regions and progression to systemic infections. Identical clones were also identified in patients and on hospital surfaces, corroborating the findings of Karami et al. (2018) [89]. Similar profiles detected across different hospitals further support patterns of regional circulation, a phenomenon also documented in large-scale studies [90]. Inter-hospital circulation highlights the potential role of patient transfer, shared healthcare networks, and environmental persistence in sustaining regional dissemination [72, 91].

ST3079 was the most prevalent sequence type and has been previously reported only once in Brazil [92]. ST111 and ST235, globally recognized high-risk clones, were also identified and are known for their wide dissemination and strong association with resistance mechanisms [12, 93]. ST277, which is endemic in Brazil and commonly associated with *blaSPM*, was also detected, although the gene was not present in the isolates analyzed [94]. The predominance of ST3079 and the detection of internationally recognized high-risk clones suggest local adaptation associated with global lineage circulation, highlighting the need for continuous molecular surveillance. The inter-hospital dissemination of these clones carries important clinical and epidemiological implications, as their persistence in moist reservoirs and biofilm-forming capacity favor environmental survival and cross-transmission in intensive care units. In addition, patient transfers and interconnected healthcare networks may facilitate their regional spread. From a clinical perspective, the persistence of these clones may compromise therapeutic effectiveness and prolong hospital stays, reinforcing the need for integrated infection control measures and rigorous environmental hygiene practices [72, 95]. Although PFGE combined with MLST provided valuable epidemiological insights into clonal dissemination, these approaches do not capture fine-scale genomic variation. Whole-genome sequencing offers higher resolution for phylogenomic analysis and enables identification of chromosomal resistance mechanisms, mobile genetic elements, and virulence determinants. Future studies using WGS are warranted to further elucidate the evolutionary dynamics and genomic basis of carbapenem resistance among *P. aeruginosa* circulating in the Western Amazon region.

The inter-hospital dissemination and transmission between patients and the hospital environment observed for dominant clones suggest the presence of lineages with high adaptive capacity and persistence in the healthcare setting [72]. Successful *Pseudomonas aeruginosa* clones frequently combine multidrug resistance with the acquisition of mobile genetic determinants, features that enhance antimicrobial tolerance and promote survival under therapeutic pressure [71, 95]. In addition, biofilm-forming capacity facilitates survival on inert surfaces and promotes cross-transmission in critical care units, contributing to environmental persistence and colonization of medical devices and hospital reservoirs [69]. Collectively, these characteristics confer an ecological advantage to high-risk clones and underscore the importance of integrated genomic surveillance for the control of healthcare-associated infections [72].

Taken together, the results demonstrate high levels of multidrug resistance, widespread distribution of β-lactamases, and a strong capacity for biofilm formation, coupled with clonal circulation among patients, hospital surfaces, and different hospitals. These findings underscore the urgent need for robust molecular surveillance strategies, infection control measures, and rational antimicrobial use. Targeted interventions should include strengthened antimicrobial stewardship programs, enhanced environmental disinfection protocols, routine molecular surveillance of high-risk clones, and interinstitutional communication to mitigate regional spread. 

## Supplementary Information

Below is the link to the electronic supplementary material.

Supplementary Material 1 (PDF 178 KB)

Supplementary Material 2 (PDF 182 KB)

Supplementary Material 3 (PDF 177 KB)

## Data Availability

Datasets related to this article will be available upon request to the corresponding author.
