# Supplementary material for: Multidrug-resistant Pseudomonas aeruginosa in ICU patients and hospital surfaces: β-lactamase burden, biofilm formation and clonal spread
Source: Eur J Clin Microbiol Infect Dis. 2026 Mar 21;45(7):1979–94. doi: 10.1007/s10096-026-05457-w (PMC13328141; doi:10.1007/s10096-026-05457-w)
Supplement: Supplementary file 1 — Supplementary Material 1 (PDF 178 KB) [file 10096_2026_5457_MOESM1_ESM.pdf]

Multidrug-Resistant *Pseudomonas aeruginosa* in ICU Patients and Hospital Surfaces: β-Lactamase Burden, Biofilm Formation and Clonal Spread

Marcos Eduardo Passos da Silva[a,b] (0000-0002-4973-0181); Luccas Manoel de Melo Suica[a] (0009-0004-2135-1368) ; Renata Santos Rodrigues[a,c] (0000-0001-7954-864X) ; Márlon Grégori Flôres Custódio[e](0000-0002-5700-1923); Valcimar Batista Ferreira[a] (0009-0008-8362-3049); Leilane da Silva Pontes[d] (0009-0005-5240-2241); Ivson Cassiano de Oliveira Santos[d] (0000-0001-9909-1405); Bruno Rocha Pribul[d] (0000-0001-9891-0616); Núcia Cristiane da Silva Lima[a,c] (0000-0001-8588-3188) ; Izabelly Vitória Gotara Ramos[a] (0009-0001-2452-8569); Anjo Gabriel Carvalho[a,b] (0000-0002-1870-0465); Mayra Gyovana Leita Belém[a] (0000-0002-1801-4115); Rosimar Pires Esquerdo[a] (0009-0007-3227-4662); Ana Paula D'Alincourt Carvalho Assef[d] (0000-0001-7044-4596); Najla Benevides Matos[a,b] (0000-0002-7271-5764).

[a]Oswaldo Cruz Foundation – Rondônia, Porto Velho, Rondônia, Brazil;

[b]Federal University of Rondônia, Experimental Biology Post-Graduate Program (PGBIOEXP), Porto Velho, Rondônia, Brazil;

[c]Tropical Medicine Research Center (CEPEM), Porto Velho, Rondônia, Brazil.

[d]Hospital Infection Research Laboratory (LAPIH), Oswaldo Cruz Institute (IOC), Rio de Janeiro, Rio de Janeiro, Brazil.

[e]Evandro Chagas National Institute of Infectious Diseases – INI/FIOCRUZ

Corresponding author: Marcos Eduardo Passos da Silva

E-mail: marcos.passos@fiocruz.br; marcoseduardo48@gmail.com

Supplementaty Material 1 Reference strains used as positive and negative controls for PCR reactions

**Genes presents in the strain**	**Reference strains**
blaCTX-M	*Klebsiella pneumoniae* CCBH 6556
blaSHV	*Klebsiella pneumoniae* CCBH 6556
blaTEM	*Klebsiella pneumoniae* CCBH 6556
blaGES	*Pseudomonas aeruginosa* CCBH 28850
blaKPC	*Klebsiella pneumoniae* CCBH 6556
blaNDM	*Klebsiella pneumoniae* CCBH 16302
blaOXA-48	*Klebsiella pneumoniae* CCBH 18079
blaIMP	*Pseudomonas aeruginosa* CCBH 24808
blaSPM	*Pseudomonas aeruginosa* CCBH 23483
blaVIM	*Pseudomonas aeruginosa* CCBH 27919
mcr-1	*Escherichia coli* CCBH 20180
Negative control	*Escherichia coli* ATCC 25922
