# Supplementary material for: Multidrug-resistant Pseudomonas aeruginosa in ICU patients and hospital surfaces: β-lactamase burden, biofilm formation and clonal spread
Source: Eur J Clin Microbiol Infect Dis. 2026 Mar 21;45(7):1979–94. doi: 10.1007/s10096-026-05457-w (PMC13328141; doi:10.1007/s10096-026-05457-w)
Supplement: Supplementary file 2 — Supplementary Material 2 (PDF 182 KB) [file 10096_2026_5457_MOESM2_ESM.pdf]

Multidrug-Resistant *Pseudomonas aeruginosa* in ICU Patients and Hospital Surfaces: β-Lactamase Burden, Biofilm Formation and Clonal Spread

Marcos Eduardo Passos da Silva[a,b] (0000-0002-4973-0181); Luccas Manoel de Melo Suica[a] (0009-0004-2135-1368) ; Renata Santos Rodrigues[a,c] (0000-0001-7954-864X) ; Márlon Grégori Flôres Custódio[e](0000-0002-5700-1923); Valcimar Batista Ferreira[a] (0009-0008-8362-3049); Leilane da Silva Pontes[d] (0009-0005-5240-2241); Ivson Cassiano de Oliveira Santos[d] (0000-0001-9909-1405); Bruno Rocha Pribul[d] (0000-0001-9891-0616); Núcia Cristiane da Silva Lima[a,c] (0000-0001-8588-3188) ; Izabelly Vitória Gotara Ramos[a] (0009-0001-2452-8569); Anjo Gabriel Carvalho[a,b] (0000-0002-1870-0465); Mayra Gyovana Leita Belém[a] (0000-0002-1801-4115); Rosimar Pires Esquerdo[a] (0009-0007-3227-4662); Ana Paula D'Alincourt Carvalho Assef[d] (0000-0001-7044-4596); Najla Benevides Matos[a,b] (0000-0002-7271-5764).

[a]Oswaldo Cruz Foundation – Rondônia, Porto Velho, Rondônia, Brazil;

[b]Federal University of Rondônia, Experimental Biology Post-Graduate Program (PGBIOEXP), Porto Velho, Rondônia, Brazil;

[c]Tropical Medicine Research Center (CEPEM), Porto Velho, Rondônia, Brazil.

[d]Hospital Infection Research Laboratory (LAPIH), Oswaldo Cruz Institute (IOC), Rio de Janeiro, Rio de Janeiro, Brazil.

[e]Evandro Chagas National Institute of Infectious Diseases – INI/FIOCRUZ

Corresponding author: Marcos Eduardo Passos da Silva

E-mail: marcos.passos@fiocruz.br; marcoseduardo48@gmail.com

Supplementaty material 2 PCR conditions used for the amplification of resistance genes

Gene	Reagents/conditions*	Cycling parameters
blaCTX-M	4.9 µL ultrapure H_2O; 1 µL 10X Buffer; 1 µL dNTPs 2.5 mM; 1 µL $MgCl_2$ 50 mM; 1 µL primers (10 pmol); 0.1 µL Taq (5 U/µL); 1 µL template DNA.	One cycle at 94°C for 2 min; 35 cycles at 95°C for 20 s, 54°C for 30 s, 72°C for 30 s; final extension at 72°C for 3 min.
blaSHV	4.2 µL ultrapure H_2O; 1 µL 10X Buffer; 1 µL dNTPs 2.5 mM; 0.6 µL $MgCl_2$ 50 mM; 2 µL primers (10 pmol); 0.2 µL Taq (5 U/µL); 1 µL template DNA.	One cycle at 95°C for 3 min; 30 cycles at 95°C for 30 s, 55°C for 30 s, 72°C for 30 s; final extension at 72°C for 3 min.
blaTEM-1	5.4 µL ultrapure H_2O; 1 µL 10X Buffer; 1 µL dNTPs 2.5 mM; 0.5 µL $MgCl_2$ 50 mM; 1 µL primers (10 pmol); 0.1 µL Taq (5 U/µL); 1 µL template DNA.	One cycle at 95°C for 3 min; 30 cycles at 95°C for 1 min, 42°C for 1 min, 72°C for 1 min; final extension at 72°C for 3 min.
blaGES	6.95 µL ultrapure H_2O; 1 µL 10X Buffer + $MgCl_2$ 50 mM; 1 µL dNTPs 2.5 mM; 0.2 µL primers (10 pmol); 0.25 µL Taq (5 U/µL); 1 µL template DNA.	One cycle at 95°C for 5 min; 35 cycles at 95°C for 20 s, 62°C for 45 s, 72°C for 30 s; final extension at 72°C for 5 min.
blaKPC	6.1 µL ultrapure H_2O; 1 µL 10X Buffer; 1 µL dNTPs 2.5 mM; 0.4 µL $MgCl_2$ 50 mM; 0.2 µL primers (10 pmol); 0.1 µL Taq (5 U/µL); 1 µL template DNA.	One cycle at 95°C for 5 min; 35 cycles at 95°C for 20 s, 60°C for 40 s, 72°C for 30 s; final extension at 72°C for 5 min.
blaNDM	5.7 µL ultrapure H_2O; 1 µL 10X Buffer; 1 µL dNTPs 2.5 mM; 0.8 µL $MgCl_2$ 25 mM; 0.2 µL primers (10 pmol); 0.1 µL Taq (5 U/µL); 1 µL template DNA.	One cycle at 95°C for 5 min; 35 cycles at 95°C for 20 s, 60°C for 40 s, 72°C for 30 s; final extension at 72°C for 5 min.
blaOXA-48	3.9 µL ultrapure H_2O; 1 µL 10X Buffer; 1 µL dNTPs 2.5 mM; 1 µL $MgCl_2$ 25 mM; 2 µL primers (10 pmol); 0.1 µL Taq (5 U/µL); 1 µL template DNA.	One cycle at 95°C for 5 min; 35 cycles at 95°C for 20 s, 60°C for 40 s, 72°C for 30 s; final extension at 72°C for 5 min.
blaIMP-1	3.9 µL ultrapure H_2O; 1 µL 10X Buffer; 1 µL dNTPs 2.5 mM; 1 µL $MgCl_2$ 25 mM; 1 µL primers (10 pmol); 0.1 µL Taq (5 U/µL); 1 µL template DNA.	One cycle at 94°C for 5 min; 30 cycles at 94°C for 45 s, 62°C for 35 s, 72°C for 1 min; final extension at 72°C for 5 min.
blaSPM	3.9 µL ultrapure H_2O; 1 µL 10X Buffer; 1 µL dNTPs 2.5 mM; 1 µL $MgCl_2$ 25 mM; 1 µL primers (10 pmol); 0.1 µL Taq (5 U/µL); 1 µL template DNA.	One cycle at 94°C for 5 min; 30 cycles at 94°C for 45 s, 62°C for 35 s, 72°C for 1 min; final extension at 72°C for 5 min.

blaVIM	6.95 μL ultrapure H_2O; 1 μL 10X Buffer + $MgCl_2$ 50 mM; 1 μL dNTPs 2.5 mM; 0.2 μL primers (10 pmol); 0.25 μL Taq (5 U/μL); 1 μL template DNA.	One cycle at 95°C for 5 min; 35 cycles at 95°C for 20 s, 62°C for 45 s, 72°C for 30 s; final extension at 72°C for 5 min.
mcr-1	4.9 μL ultrapure H_2O; 1 μL 10X Buffer; 1 μL dNTPs 2.5 mM; 1 μL $MgCl_2$ 50 mM; 1 μL primers (10 pmol); 0.1 μL Taq (5 U/μL); 1 μL template DNA.	One cycle at 94°C for 5 min; 30 cycles at 94°C for 45 s, 60°C for 45 s, 72°C for 45 s; final extension at 72°C for 5 min.

*All reactions were performed at a final volume of 10 μL.
