# Supplementary material for: Multidrug-resistant Pseudomonas aeruginosa in ICU patients and hospital surfaces: β-lactamase burden, biofilm formation and clonal spread
Source: Eur J Clin Microbiol Infect Dis. 2026 Mar 21;45(7):1979–94. doi: 10.1007/s10096-026-05457-w (PMC13328141; doi:10.1007/s10096-026-05457-w)
Supplement: Supplementary file 3 — Supplementary Material 3 (PDF 177 KB) [file 10096_2026_5457_MOESM3_ESM.pdf]

Multidrug-Resistant *Pseudomonas aeruginosa* in ICU Patients and Hospital Surfaces: β-Lactamase Burden, Biofilm Formation and Clonal Spread

Marcos Eduardo Passos da Silva[a,b] (0000-0002-4973-0181); Luccas Manoel de Melo Suica[a] (0009-0004-2135-1368) ; Renata Santos Rodrigues[a,c] (0000-0001-7954-864X) ; Márlon Grégori Flôres Custódio[e](0000-0002-5700-1923); Valcimar Batista Ferreira[a] (0009-0008-8362-3049); Leilane da Silva Pontes[d] (0009-0005-5240-2241); Ivson Cassiano de Oliveira Santos[d] (0000-0001-9909-1405); Bruno Rocha Pribul[d] (0000-0001-9891-0616); Núcia Cristiane da Silva Lima[a,c] (0000-0001-8588-3188) ; Izabelly Vitória Gotara Ramos[a] (0009-0001-2452-8569); Anjo Gabriel Carvalho[a,b] (0000-0002-1870-0465); Mayra Gyovana Leita Belém[a] (0000-0002-1801-4115); Rosimar Pires Esquerdo[a] (0009-0007-3227-4662); Ana Paula D'Alincourt Carvalho Assef[d] (0000-0001-7044-4596); Najla Benevides Matos[a,b] (0000-0002-7271-5764).

[a]Oswaldo Cruz Foundation – Rondônia, Porto Velho, Rondônia, Brazil;

[b]Federal University of Rondônia, Experimental Biology Post-Graduate Program (PGBIOEXP), Porto Velho, Rondônia, Brazil;

[c]Tropical Medicine Research Center (CEPEM), Porto Velho, Rondônia, Brazil.

[d]Hospital Infection Research Laboratory (LAPIH), Oswaldo Cruz Institute (IOC), Rio de Janeiro, Rio de Janeiro, Brazil.

[e]Evandro Chagas National Institute of Infectious Diseases – INI/FIOCRUZ

Corresponding author: Marcos Eduardo Passos da Silva

E-mail: marcos.passos@fiocruz.br; marcoseduardo48@gmail.com

Supplementary material 3 Sequence types, allelic profiles, and clonal complexes identified in *Pseudomonas aeruginosa* isolates from hospitalized patients and hospital surfaces

**No. of isolates**	**ST**	**CC**	**Allelic profile**						
			acsA	***aroE***	***guaA***	***mutL***	***nuoD***	***ppsA***	***trpE***
1	111	111	17	5	5	4	4	4	3
2	227	235	38	11	3	9	1	2	4
1	235	235	38	11	3	13	1	2	4
2	244	244	17	5	12	3	14	4	7
1	277	277	39	5	9	11	27	5	2
1	313	313	47	8	7	6	8	11	40
1	532	532	5	4	5	5	5	20	4
1	651	235	38	11	3	13	3	2	4
1	712	712	31	12	65	19	13	7	23
2	796	3854	36	5	1	3	2	6	1
8	3079	3178	28;74*	286	12	3	1;3;27*	4	185
1	3972	155	36	5	12	3	3	13	7
1	4864	277	39	5	9	11	4	5	2
1	4865	244	17	5	12	3	3	4	7
1	4866	274	23	5	11	7	14	12	7
1	4867	244	307	5	12	3	14	4	7

ST: Sequence Type; CC: Clonal Complex; * = different alleles encoding the same gene.
